# Systematically lower aEEG amplitude values in neurologically healthy children using an automated algorithm compared to a semi-manual method

**DOI:** 10.3389/fneur.2025.1562580

**Published:** 2025-10-08

**Authors:** Sandra Greve, Pia Brensing, Luisa Paul, Christian Dohna-Schwake, Julia Wichlacz, Ursula Felderhoff-Mueser, Nora Bruns

**Affiliations:** ^1^Department of Pediatrics I, Neonatology, Pediatric Intensive Care, Pediatric Infectious Diseases and Pediatric Neurology, University Hospital Essen, University of Duisburg-Essen, Essen, Germany; ^2^Center for Translational Neuro- and Behavioral Sciences (C-TNBS), University Hospital Essen, University of Duisburg-Essen, Essen, Germany; ^3^Department of Pediatric Cardiology/Congenital Cardiology, Heidelberg University Medical Center, Heidelberg, Germany

**Keywords:** amplitude-integrated EEG, neuromonitoring, pediatric intensive care, reference values, children, infants

## Abstract

**Objective:**

The objective of this study was to compare different modes of amplitude-integrated EEG (aEEG) assessment (semi-manual vs. automated) in children.

**Methods:**

A total of 450 unremarkable pediatric EEGs from children aged 6 months to 17.9 years were converted into aEEGs and the medians and means of the upper and lower amplitudes (C3–P3, C4–P4, C3–C4, P3–P4, Fp1–Fp2) were determined. The agreement of the semi-manual and automated measurements was assessed via the Pearson correlation coefficients (PCC) and Bland-Altman plots. Mean differences between the methods and age-specific percentiles (5th−95th) were calculated.

**Results:**

Semi-manually measured amplitudes were systematically greater than automated assessments. Mean differences of the means ranged between 23.7 and 29.3 μV for the upper and between 2.4 and 4.4 μV for the lower amplitudes depending on the channel. The PCC ranged between 0.68 and 0.92 for the upper and lower amplitudes of the mean depending on the channel. Age-specific percentiles showed different absolute values but similar trends.

**Conclusion:**

AEEG amplitude values systematically differ between semi-manual and automated assessment. Age-related trends are evident despite differences in the absolute values. Reference values for different measurement techniques are needed for pediatric aEEG.

## Introduction

Amplitude-integrated EEG (aEEG), a simple form of quantitative EEG, is increasingly spreading from neonatology to pediatric intensive care because of its accesibility. It is used as a real-time bedside tool for long-term monitoring, detection of seizures and changes in electrocortical function. Reference values of aEEG for children have rather recently been published (1, 2, 13–15). However, the comparability of measured amplitudes between different assessment methods has not been investigated.

In classic cerebral function monitoring, the aEEG is generated from a 1 or 2 channel EEG. The height of the amplitude is plotted semi-logarithmically on the y-axis, whereas the x-axis is displayed in a time-compressed manner compared to conventional EEG, with one display width resembling 3–4 h. The height, width and density of the resulting characteristic band changes depending on the electrocortical activity (1).

Different producers of aEEG devices use similar but slightly different algorithms to obtain the aEEG, with some manufacturers offering customizable processing options. For that reason, the obtained aEEG bands may vary between manufacturers, even though the overall interpretation (e.g., occurrence of seizures, classification of background patterns, etc.) (2) seems to be unaffected by these differences in neonates.

The aEEG can be assessed in various ways. The most common approach is visual bedside analysis of the obtained aEEG band and its changes over time. Suspicious sections can be viewed in detail by specialists by specifically addressing the sections of interest with a review of the raw EEG curve. The increase in amplitude is typically assessed visually and not measured (manually). Some manufacturers provide semi-manual analysis, in which the amplitude level is calculated/measured, but interpretations must be made by a reviewer. Another option is fully automated analyses, which calculate the amplitude heights independently. These methods also enable interpretation algorithms, such as spike detection. Seizures are detected automatically by analyzing the background pattern via waveform morphology and voltage field propagation (3). These methods of aEEG amplitude assessment yield different results that have implications for clinical decision making. Two studies on normal values, one that used automated and one used semi-manual assessment in healthy children reported different absolute amplitude values, whereas the same trends with respect to age (4, 5) and differences between sleep and wakefulness were observed (1, 13).

The aim of this study was to investigate the agreement of amplitude values and age-related trends from fully-automated aEEG assessment vs. semi-manually measured values in normal EEGs from neurologically healthy children. For this purpose, we conducted automated amplitude assessment of 450 EEGs with previously derived reference values for children via a semi-manual assessment (5).

## Methods

### aEEG processing

Full-channel EEGs were conducted according to the international 10–20 system. All EEGs were recorded using Neurofax EEG devices and Polaris.one software v4.0.4.0 (Nihon Kohden, Tokyo, Japan). To generate an aEEG from the raw EEG, the signal was amplified, band-pass filtered, logarithmically transformed, and finally rectified (6) using two different softwares by two manufacturers (Polaris.one software v4.0.4.0, Nihon Kohden, Tokyo, Japan and Persyst 13, PERSYST DEVELOPMENT CORPORATION, Solana Beach, USA). The exact algorithms used to generate the aEEG in both Polaris (semi-manual amplitude assessment) and Persyst (automated amplitude assessment) have not been disclosed by the providers, which is a well-known issue among different aEEG providers (2, 7). All EEGs were found to be normal by a board-certified pediatric neurologist (ADM) with additional certificates in EEG and epileptology by the German Society for Epileptology (DGfE).

### Original study (semi-manual amplitude assessment)

For the original study, unremarkable EEGs from neurologically healthy, awake children without neuroactive medication aged between 1 month and 17 years were included (213 females, 237 males) (Table 1). These EEGs were converted to aEEG using Polaris software. The filter was set at 70 Herz (Hz), the sensitivity at 7 uV/mm and the time constant at 0,3s. Amplitude values of the upper and lower amplitudes of the C3–P3, C4–P4, C3–C4, P3–P4 and Fp1–Fp2 channels of the 10–20 system were measured semi-manually with an integrated software tool by two independent investigators and age-related percentiles were calculated. Interrater reliability was presented in the original paper using Bland-Altman plots and the intraclass correlation coefficient (ICC). For the upper and lower borders, the ICC was 3.1. (5).

**Table 1:** Included patients, as previously published (5).

**Gender**	***n* (%)**
**Male**	**237 (52.7)**
**Age group [years]**	
<1	44 (9.8)
1	30 (6.7)
2–4	96 (21.3)
6–9	79 (17.6)
10–13	86 (19.1)
14–17	115 (25.6)
**Indications**	
Routine diagnostics^*^	273 (60.7)
Diagnostic work up in patients with suspected inborn or acquired neurologic disease^**^	177 (39.3)

^*^Before initiation of therapy/during aftercare, e.g., before or after solid organ transplant, bone marrow transplant, or chemotherapy; ^**^No neurologic disease diagnosed after completion of diagnostics at any timepoint in the patient history (inborn) or before the recording (acquired).

### Automated amplitude assessment

**Figure 1:**
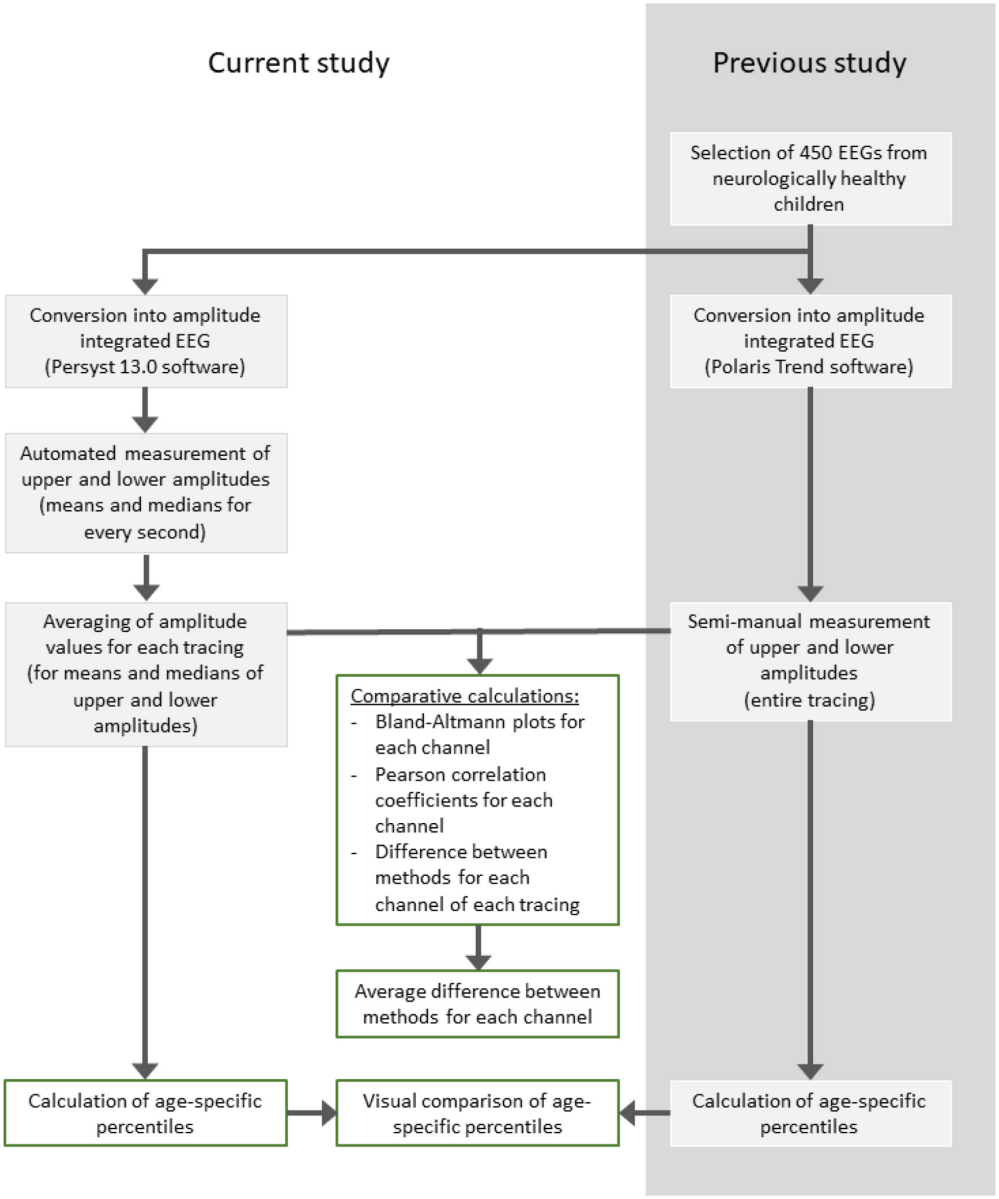
Flow chart of aEEG assessment.

For this study, automated amplitude assessment was conducted from the same EEGs and for the same channels with Persyst software. The entire recording was analyzed without artifact reduction, a high pass filter at 35 Hz, a notch filter at 50–60 Hz and the time constant at 0.1 s. The maximum (upper amplitude) and minimum (lower amplitude) margin values were calculated for each second of the recording and exported to.csv files. Next, the upper and lower amplitudes of each channel were summarized as the mean and median across the recording (Figure 1).

### Statistical analyses

All subsequently described analyses were conducted separately for the upper and lower amplitudes of each channel. We calculated Pearson correlation coefficients to assess the relationship between the semi-manually measured amplitudes from the previous study and the newly calculated mean and median amplitudes from this study. Bland-Altmann plots were generated to visually assess the agreement of the two measurement methods for each channel. For each EEG, the mean difference between the two methods was calculated for the upper and lower amplitudes of each channel.

Based on the results of the automated analyses, we calculated percentiles (5th, 10th, 25th, 50th, 75th, 90th, 95th) grouped by age because the observed differences between the semi-manual and automated assessments were considered relevant for interpretation in daily clinical practice. Because the correlation coefficients were greater for the medians of the upper and lower amplitudes than for the means, the medians were less sensitive to outliers. In addition, since MacDarby et al. also used medians for their calculations, we calculated the percentiles from medians (4).

### Ethics statement

The studies involving human participants were reviewed and approved by Ethics Committee of the Medical Faculty of the University of Duisburg-Essen (20–9444- BO). Informed consent was not necessary according to local legislation because retrospective anonymized data were used. This study was performed according to the principles of the Declaration of Helsinki.

## Results

Four hundred-fifty normal EEGs from neurologically healthy children were analyzed. The median recording time was 17 (interquartile range (IQR) 15–18) min. 52.7% of the patients were male. All EEGs were derived from children without neurological findings (Table 1).

The correlation coefficients ranged between 0.92 and 0.95 for the median and between 0.68 and 0.84 for the mean of the upper amplitudes with variations between channels (Table 2). For the lower amplitude, the correlation coefficients ranged between 0.94 and 0.98 for the median and between 0.86 and 0.92 for the mean (Table 2).

**Table 2:** Mean differences and correlation coefficients between manual and automated assessment for median and mean upper and lower amplitudes by channel; SD, standard deviation.

		**Median amplitudes per recording**		**Mean amplitudes per recording**	
**Channel**	**Amplitude**	**Mean difference** ±**SD**	**Correlation coefficient**	**Mean difference** ±**SD**	**Correlation coefficient**
P3_P4	Upper	35.2 ± 13.7	0.92	32.6 ± 14.4	0.79
C3_C4		31.9 ± 12.2	0.95	29.3 ± 13.9	0.69
C3_P3		26.8 ± 12.1	0.92	24.3 ± 13.5	0.68
C4_P4		26.2 ± 10.2	0.95	23.7 ± 11.5	0.74
FP1_FP2		35.8 ± 20.6	0.93	30.3 ± 18.0	0.84
P3_P4	Lower	5.3 ± 2.4	0.97	4.4 ± 3.0	0.92
C3_C4		5.0 ± 1.9	0.98	4.1 ± 3.0	0.88
C3_P3		4.0 ± 1.8	0.97	3.1 ± 2.7	0.90
C4_P4		3.9 ± 1.8	0.97	3.1 ± 2.9	0.88
FP1_FP2		4.1 ± 2.0	0.94	2.4 ± 2.8	0.86

Bland-Altmann plots revealedsystematic deviations between the measurement methods, with higher values obtained from manual measurements. The mean differences in the medians between the two methods ranged between 26.2 and 35.8 for the upper amplitudes and between 3.9 and 5.3 for the lower amplitudes. The mean differences in the means between the two methods ranged between 23.7 and 29.3 μV for the upper amplitudes and between 2.4 and 4.4 for the lower amplitudes (Table 2).

**Figure 2:**
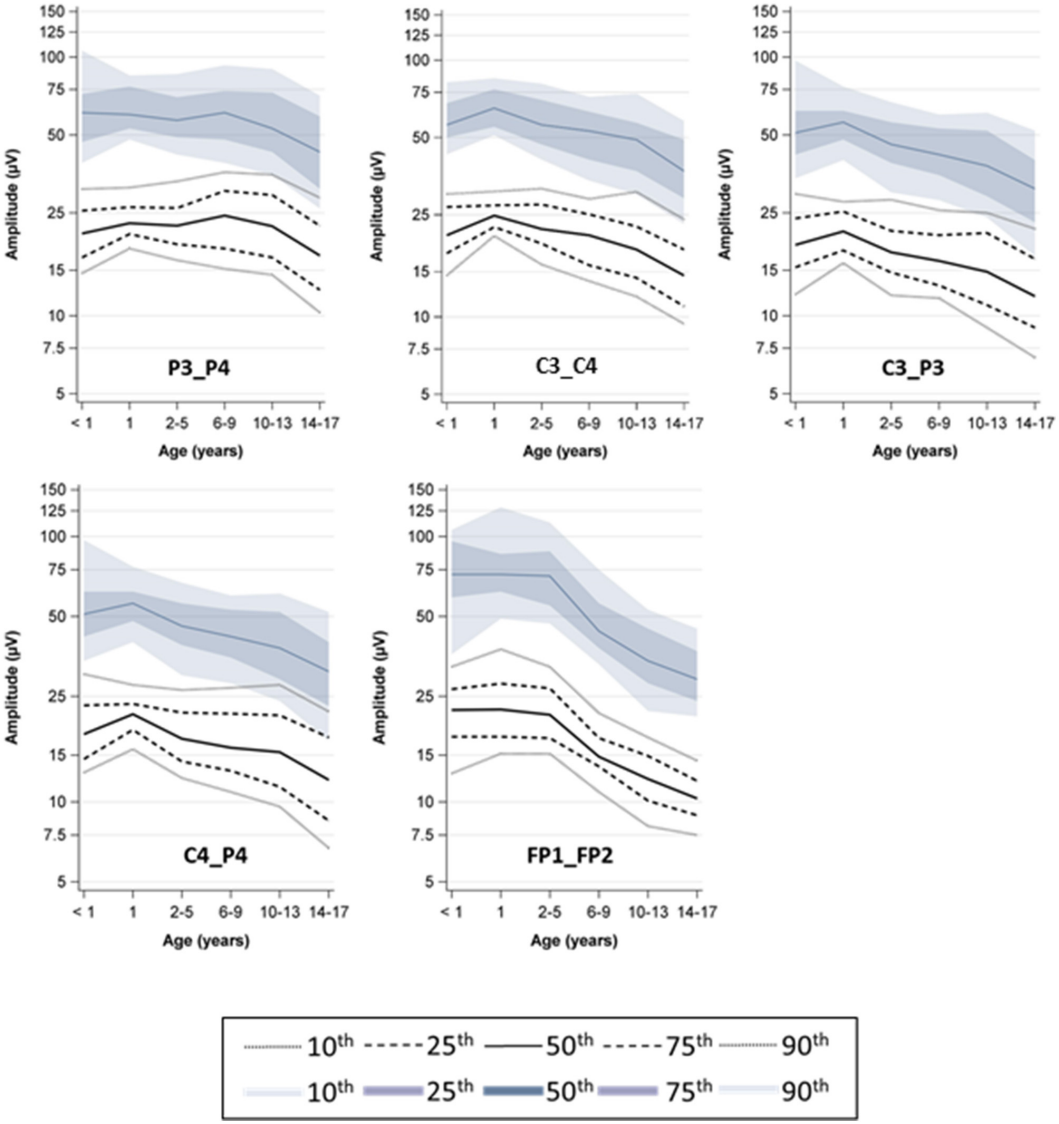
Percentile curves by age, upper boarder; the colored graphs show the semi-manual measurement (previous study), the dashed graphs show the automated measurement (current study).

**Figure 3:**
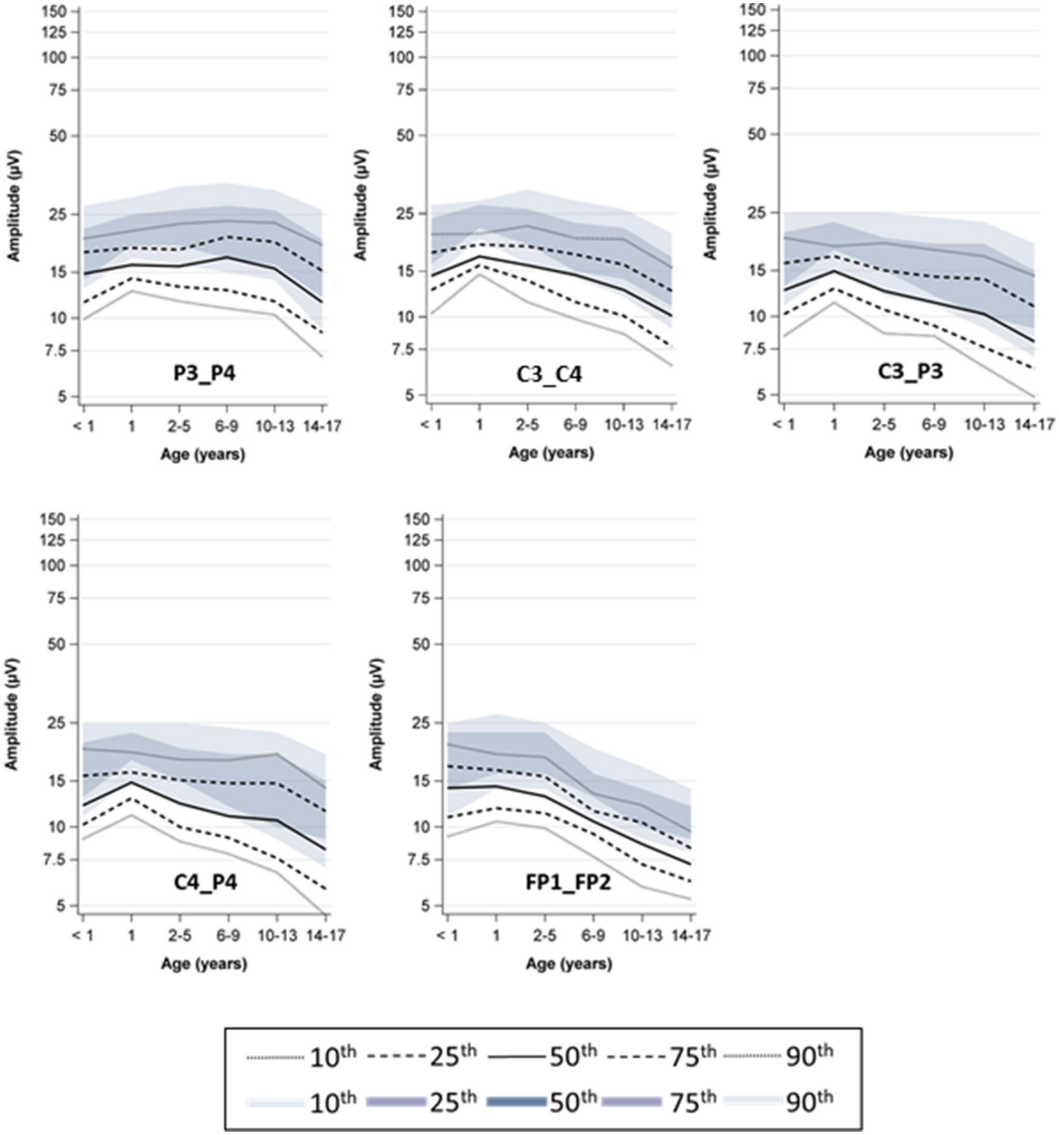
Percentile curves by age, lower boarder; the colored graphs show the semi-manual measurement (previous study), the dashed graphs show the automated measurement (current study).

The age-specific percentiles (5th−95th percentile) from automated assessment showed a similar age dependency as previously published, but with different absolute values (Figures 2, 3).

## Discussion

This study compared semi-manual with fully automated aEEG amplitude assessment and detected systematically lower values for automated assessment of upper and lower aEEG amplitudes, whereas age-related trends corresponded to those observed via semi-manual assessment. The percentiles derived from the automated assessment were consistently lower compared than those derived fromthe semi-manual measurement. These findings indicate that different analysis methods produce strong variations in amplitude values, making it necessary to adapt reference values and amplitude-interpretations to the specific techniques.

Previous evidence on aEEG reference values in children is scarce. Compared to our previous semi-manual assessment (5), MacDarby et al. reported lower values for the upper amplitudes for C-P channels. In contrast, the determined values for the lower amplitude were higher (4). Besides the different methods, the MacDarby study analyzed a 5-min artifact-free section. Furthermore, children with epilepsy who werereceiving neuroactive drugs such as anticonvulsants were included. Different processing algorithms between manufacturers may also have produced slightly different results. However, the results from our presented automated assessment that used the same algorithm as MacDarby et al. indicated strikingly similar values for both the upper and the lower amplitudes.

In addition to analysis techniques, differing processing algorithms between manufacturers may also cause variations in theobtained aEEG amplitudes (8). Sabir and Hoehn reported that different aEEG devices generate different absolute values at upper voltage levels but that abnormalities are detected in a comparable manner, suggesting that there isno difference in treatment decisions (2). These findings justify the currently practiced bedside interpretation of neonatal aEEG using mainly manual/visual classifications, e.g. by Hellström-Westas, Burdjalov or Olischar (9–11). However, ongoing digitalization expands the possibilities for automated assessment and makes knowledge about differences between measurement techniques and processing algorithms important for interpretation. This is of particular significance when switching between manual/visual assessment and automated algorithms. Given that aEEG producers do not publicly provide their processing and transformation algorithms, open-source algorithms may become necessary to increase comparability and reproducibility between devices. Further, reference values for various measurement methods should be established, as already postulated by MacDarby (4).

An important limitation of automated EEG assessment is the software's ignorance of the clinical context. Thus, assessment is carried out without taking the particular clinical setting and patient's situation into account. In manual or semi-manual interpretation, however, these sections can be ignored more easily to “blind out” interfering factors and potential artifacts. Furthermore, automated artifact removal typically uses blind source separation (BSS), as in Persyst software (3). By this means, removal of artifacts can cause loss of information if neuronal information is superimposed by interfering information (12).

There are several limitations to our study. First, we used different software packages to transform the raw EEGs into aEEGs for semi-manual and automated assessment, providing the first opportunity for diverging results. However, the initial software does not support automated export of values, and Persyst does not provide an option for manual measurement. Second, artifact reduction was turned off for the automatic assessment, because otherwise entire recordings would have become unavailable. Importantly, this shortcoming of artifact removal software also applies to aEEG processing in the clinical setting, possibly making thevalues provided here more real-world proof than completely “cleaned” values. Third, analyzed recordings were rather short, enhancing the potential impact of transient amplitude changes on the obtained values. This limitation, however, applies only to the absolute reference values but not for the comparison between analysis methods.

## Conclusion

Upper and lower aEEG amplitude values in children systematically differ between semi-manual and automated measurements. Theresults obtained from both methods were congruent regarding age-related trends and differences between electrode positions, but clearly highlightthe need to establish reference values for each technique and potentially even for each different processing algorithm. In any case, clinicians must pay close attention to the mode of measurement when interpreting aEEG amplitude values.

## Data Availability

The raw data supporting the conclusions of this article will be made available by the authors, without undue reservation.
